# Insights into the Interactions of *Fasciola hepatica* Cathepsin L3 with a Substrate and Potential Novel Inhibitors through *In Silico* Approaches

**DOI:** 10.1371/journal.pntd.0003759

**Published:** 2015-05-15

**Authors:** Lilian Hernández Alvarez, Dany Naranjo Feliciano, Jorge Enrique Hernández González, Rosemberg de Oliveira Soares, Diego Enry Barreto Gomes, Pedro Geraldo Pascutti

**Affiliations:** 1 Departamento de Biología Molecular, Centro Nacional de Sanidad Agropecuaria de Cuba (CENSA), San José de las Lajas, Mayabeque, Cuba; 2 Centro de Estudios de Proteínas (CEP), Facultad de Biología, Universidad de la Habana, La Habana, Cuba; 3 Instituto de Biofísica Carlos Chagas Filho, Universidade Federal do Rio de Janeiro (UFRJ), Rio de Janeiro, Brazil; 4 Diretoria de Metrologia Aplicada às Ciências da Vida (DIMAV), Instituto Nacional de Metrologia, Qualidade e Tecnologia (INMETRO), Rio de Janeiro, Brazil; McGill University, CANADA

## Abstract

**Background:**

*Fasciola hepatica* is the causative agent of fascioliasis, a disease affecting grazing animals, causing economic losses in global agriculture and currently being an important human zoonosis. Overuse of chemotherapeutics against fascioliasis has increased the populations of drug resistant parasites. *F*. *hepatica* cathepsin L3 is a protease that plays important roles during the life cycle of fluke. Due to its particular collagenolytic activity it is considered an attractive target against the infective phase of *F*. *hepatica*.

**Methodology/Principal Findings:**

Starting with a three dimensional model of FhCL3 we performed a structure-based design of novel inhibitors through a computational study that combined virtual screening, molecular dynamics simulations, and binding free energy (ΔG_bind_) calculations. Virtual screening was carried out by docking inhibitors obtained from the MYBRIDGE-HitFinder database inside FhCL3 and human cathepsin L substrate-binding sites. On the basis of dock-scores, five compounds were predicted as selective inhibitors of FhCL3. Molecular dynamic simulations were performed and, subsequently, an end-point method was employed to predict ΔG_bind_ values. Two compounds with the best ΔG_bind_ values (-10.68 kcal/mol and -7.16 kcal/mol), comparable to that of the positive control (-10.55 kcal/mol), were identified. A similar approach was followed to structurally and energetically characterize the interface of FhCL3 in complex with a peptidic substrate. Finally, through pair-wise and per-residue free energy decomposition we identified residues that are critical for the substrate/ligand binding and for the enzyme specificity.

**Conclusions/Significance:**

The present study is the first computer-aided drug design approach against *F*. *hepatica* cathepsins. Here we predict the principal determinants of binding of FhCL3 in complex with a natural substrate by detailed energetic characterization of protease interaction surface. We also propose novel compounds as FhCL3 inhibitors. Overall, these results will foster the future rational design of new inhibitors against FhCL3, as well as other *F*. *hepatica* cathepsins.

## Author Summary

Fascioliosis is considered an emerging disease in humans, causing important losses in global agriculture through the infection of livestock animals. The outcome of resistant parasites has increased the search for new drugs which may contribute to disease control. In recent decades, *Fasciola* cathepsins (FhCs) have been defined as the principal virulence factors of this parasite. Despite being in the same protein family, they have different specificities and, thus, distinct roles throughout the fluke life cycle. Differences in specificity have been attributed to a few variations in the sequence of key FhCs subsites. Currently, the structure-based drug design of inhibitors against *Fasciola* cathepsin Ls (FhCLs) with unknown structures is possible due to the availability of the three-dimensional structure of FhCL1. Our detailed structural analysis of the major infective juvenile enzyme (FhCL3) identifies the molecular determinants for protein binding. Also, novel potential inhibitors against FhCL3 are proposed, which might reduce host invasion and penetration processes. These compounds are predicted to interact with the binding site of the enzyme, therefore they could prevent substrate processing by competitive inhibition. The structure-based drug design strategy described here will be useful for the development of new potent and selective inhibitors against other FhCs.

## Introduction

Fascioliasis or hepatic distomatosis, caused by the food-borne trematodes *Fasciola hepatica* and *Fasciola gigantica*, is considered one of the most important parasitic diseases, which constitutes a serious public health problem and has a significant veterinary relevance. Economically important animals affected by this disease include cattle, sheep and goats [1, 2]. Fascioliasis symptoms are host-specific, but generally comprise reduced milk and wool yields, weight gains, and fertility [3]. Recently, the global burden of fascioliasis was calculated and it has been estimated that 2.6 million people are infected with *Fasciola* spp. [4].

Despite the economic losses as well as the negative impact on human health, chemotherapy is currently the only viable parasite control mechanism. Benzimidazoles, in particular triclabendazole, are the most commonly-used drugs. Their targets are both immature and mature forms of the parasite, but their continued use has led to drug resistance [5]. Therefore, the search for new strategies and target molecules for the development of novel fasciolicide drugs is urgently required.

The most abundant molecules found in *F*. *hepatica* secretions are papain-like cysteine proteases, termed cathepsins, which are grouped in cathepsin L and B families [6, 7]. They are secreted in vesicle packages by gastrodermal cells into parasite gut lumen, and then released into host tissues [8]. In recent decades, the role of these proteases has been widely studied due to their importance as potential targets for the treatment of many parasite infections [9]. Cathepsins are critical for the development and survival of the parasite within the mammalian hosts. They participate in the digestion of host components such as fibronectin, collagen and albumin, which facilitates parasite migration and feeding, and can also degrade immunoglobulins and T cell surface molecules, thereby promoting immune evasion [10–12]. These proteases have an active site formed by five subsites, i.e., S3-S2-S1-S1’-S2’, the substrate specificity being governed by S2 and S3 subsites [13]. An analysis of the residues comprising the S2 and S3 subsites in several members of the cathepsin L family, reveals the divergence within these subsites, in particular at positions that have the greatest influence on substrate recognition, i.e., 61, 67, 157, 158 and 205 (papain numbering) [6, 14, 15].

*F*. *hepatica* can regulate the differential expression of cathepsins during its life cycle. These expression patterns have been associated with the functional diversity of papain-like proteases [12, 16, 17]. Previous studies have detected cathepsin B (FhCB) and L3 (FhCL3) secretion in early invasive-stage parasites [18]. The prevalence of cathepsin L-like activity after excystation was observed in *in vitro* assays [19]. Also, experiments with an RNAi derived from an FhCL1 gene fragment encoding a region conserved across the cathepsin L family, led to the induction of phenotypes with abnormal motility in *F*. *hepatica* newly-excysted juveniles (NEJ) and a significant reduction of rat intestinal wall penetration [20]. The predominant cathepsin, found by proteomic analysis in the NEJ excretion/secretion products, is procathepsin L3 (proFhCL3) [21]. The zymogen form of this peptidase progressively changes to the mature enzyme during the first 48h of NEJ development, which is mainly involved in penetration and immune response evasion [18]. Additionally, partial protection against fascioliasis in rats was obtained using a recombinant form of FhCL3 [22]. These findings suggest that FhCL3 could be a potential target for new therapies against early stages of parasite infection.

It is widely accepted that the interaction patterns between enzymes and their natural substrates provide insights for drug design [23, 24]. Accordingly, some studies have been conducted to assess the substrate specificity of FhCL3, as well as the role of some enzyme residues (i.e., H63 and W69) in the substrate-binding process [25]. It was also demonstrated the strong preference of this cathepsin for Pro and Gly residues at P2 and P3 sites, respectively, through the usage of positional scanning synthetic combinatorial libraries [25]. The previous finding was linked to the FhCL3 collagenolytic activity, since type I and type II collagens possess repeating Gly-Pro-Xaa motifs [26]. To date some *in silico* modeling tools have been applied to provide structural insights into the interaction of FhCL3 with peptidic substrates, as well as to predict the affinity of the enzyme for various peptides [27]. However, no previous detailed energetic analysis of the FhCL3-substrate interactions has been performed yet. Therefore, we believe that the latter is required not only to complement previous structural analyses, but also to establish at an atomic level the nature of the interactions present at the complex interfaces, as well as to quantify their energy contribution to the binding process.

Here we carried out a thorough energetic study of the binding site of FhCL3 in complex with a peptidic substrate based on a homology model. Furthermore, an FhCL3 model and HuCatL crystal structure were used for Virtual Screening (VS) studies and compounds with higher selectivity for the former enzyme were subsequently selected according to their Autodock Vina energy-scores (S_vina_) [28]. Finally, binding affinities were estimated through MM-GBSA absolute binding free energy (ΔG_bind_) calculations [29–31] based on thermodynamic ensembles generated with molecular dynamics (MD) simulations.

## Materials and Methods

### Sequence retrieving and functional analysis

The search for FhCL3 homologues (UniProt: Q9GRW6) [32] was carried out in a non-redundant protein database using the PSI-BLAST at the NCBI server [33]. SAS [34] and MESSA [35] servers were additionally used against the Protein Data Bank (PDB) [36] to retrieve the most suitable template. Automated multiple sequence alignment (MSA) was performed with MUSCLE v3.8.31 [37] while Seaview v4.3.1 [38] and ClustalX v2.1 [39] were used to edit the MSA and to determine conserved residues. Finally, a homology model of FhCL3 was generated with Modeller v9.11 [40] using the three dimensional (3D) structure of proFhCL1 C25G [14] (PDB: 2O6X, sequence identity ~71%) as a template, in accordance with previous works [21, 25, 27].

In order to provide insights into the binding mode of peptidic substrates to FhCL3, a 3D model of this enzyme in complex with a specific peptide, ACE-AGPR↓NAA-NME, was also built. The procedure for obtaining the complex structure was the same reported by Robinson *et al* [27].

### Virtual screening

VS against the FhCL3 homology model and the HuCatL structure (PDB: 2YJC) was carried out with AutoDock Vina v4.0 software [28]. Synthetic lead compounds from HitFinder database of the Maybridge British company (http://www.mybridge.com) were selected for this study. The VS was run using software default settings, however, the number of energetically-degenerated poses was set to ten. During docking simulations, all rotatable bonds of each ligand were allowed to freely move around the bond axes, while the protein structure was kept fixed. The grid box used to define the screening was centered on the catalytic cysteine residue, i.e., C25 of FhCL3 and HuCatL, employing AutoDockTools. Box dimensions X, Y and Z were set to 16.5, 21 and 15 Å, respectively.

To identify compounds with higher specificity for FhCL3, hit selection was based on the relative affinity for FhCL3 and HuCatL obtained from ΔS_vina_ values. Furthermore, these hits were submitted to the DrugMint server [41] for the selection of drug-like compounds based on the best probability scores. The most probable pose of each compound within the FhCL3 binding site was selected by visual inspection. Some criteria taken into account for pose selection were (*i*) the number of hydrogen bonds between the compound and the enzyme residues and (*ii*) the available information for other compounds containing some similar chemical groups in complex with papain-like proteases [42, 43].

Docking protocol validation was carried out through the non-covalent re-docking of nitrile ((2S,4R)-1-[1-(4-chlorophenyl) cyclopropyl] carbonyl-4-(2-chlorophenyl) sulfonyl-N-[1-(iminomethyl) cyclopropyl] pyrrolidine-2-carboxamide), a well-known HuCatL covalent inhibitor, into the active site of this protease [44]. Nitrile structure was taken from the crystal of HuCatL-nitrile complex solved at 1.14 Å (2YJC) [44].

### Ligand parametrization

The 3D structures of the ligands were obtained from the SDF format using Babel [45]. Then, Avogadro [46] was used for ligand protonation at pH = 7.4 and for subsequent steepest-descents energy minimization using Generalized Amber Force Field (GAFF) parameters [47]. Minimized structures were then optimized at HF/6-31G* level using Gaussian 09 package [48]. Electrostatic potentials (ESPs) for the optimized structures were finally generated by single-point calculations in Gaussian 09 with HF/6-31G* method and Merz-Kollman (MK) scheme [49]. Partial atomic charges were fitted to the ESPs through the Restricted Electrostatic Potential (RESP) method [50] implemented in the Antechamber program [51]. Likewise, ligand atom types, bond and dihedral angles, atomic masses and bond lengths were obtained from GAFF using Antechamber [51].

### Energy minimization and molecular dynamics simulations

EM of free FhCL3 and all protease-ligand complexes was performed using GROMACS v4.6.3 [52] with the AMBER99SB-ILDN force field for the enzyme [53] and GAFF for the ligands. Briefly, protonation states of the FhCL3 ionizable residues were determined at pH = 7.4 by using the PDB2PQR server [54]. All systems were solvated with explicit TIP3P water molecules [55] in a dodecahedral box whose edges were placed at a minimum distance of 1 nm from the solute surface, and neutralized by replacing water molecules with Na^+^ counter ions. EM was carry out by 50000 of steepest descents steps with a tolerance of 10 kJ/(mol·nm), to relax high energy interactions and steric clashes.

Subsequently, the equilibration procedure was conducted in two steps: NVT and NPT ensembles keeping the solute heavy atoms restrained. During the 200 ps NVT equilibration, temperature was kept constant at 300 K using the velocity-rescale thermostat [56]. The subsequent 200 ps NPT equilibration was performed at a temperature of 300 K using the same temperature coupling algorithm and at a pressure of 1 bar with the Parrinello-Rahman barostat [57]. A time step of 2 fs was employed to integrate the equation of motion using the Leap-Frog algorithm [58]. Random initial velocities taken from the Maxwell-Boltzmann distribution were assigned to the atoms of each system at 300 K. Cutoff radii of 1.4 nm and 1.0 nm were established for the calculation of van der Waals and short-range electrostatic interactions, respectively. The **P**article **M**esh **E**wald (PME) method [59] was employed to handle long-range electrostatic interactions. Periodic boundary conditions were used at the boundaries of the unit cell. Neighbor lists were defined by a cutoff radius of 1.0 nm and were updated every 10 fs. All bond lengths were constrained with the LINCS method [60].

The productive run time was 130 ns and 100 ns for FhCL3-ligand and FhCL3-substrate complexes respectively. System coordinates and initial velocities were taken from the NPT simulation output. Constant temperature and pressure of 300 K and 1 bar were maintained during the simulations with the velocity-rescale thermostat [56] and the Parrinello-Rahman barostat [57], respectively. The integrator, cutoff radii, constraint algorithm etc. were identical to those used during the equilibration steps.

### Binding free energy calculation and decomposition

MM-GBSA and the Molecular Mechanics/Poisson—Boltzmann Surface Area (MM-PBSA) are computationally efficient methods for estimating the binding free energy (ΔG_bind_) of protein-ligand complexes [29–31, 61, 62]. In these methods, ΔG_bind_ is expressed through the well-known equation:

$$\Delta G_{bind}=G_{complex}-\left[G_{protein}+G_{ligand}\right]$$ (1)

Each term on the right-hand side of the former equation is expressed as follows in the MM-GBSA and MM-PBSA formalism:

$$G=E_{MM}+G_{sol}-TS=E_{MM}+\left[G_{GB/PB}+G_{SA}\right]-TS$$ (2)

E_MM,_ is the energy of the complex, the ligand or the protein in the gas phase, calculated as the sum of the internal energy (E_inter_), the van der Waals energy (E_vdw_) and the electrostatic energy (E_elec_) as expressed below:

$$E_{MM}=E_{\mathrm{int}er}+E_{vdw}+E_{elec}$$ (3)

The second term of Eq 2, ΔG_solv_, represents the free energy of solvation calculated by means of implicit-solvation models. This term is further decomposed into the sum of the polar solvation (*G*_*GB/PB*_) and the non-polar solvation (*G*_*SA*_) contributions (Eq 4)

$$G_{sol}=G_{pol}+G_{np}=G_{GB/PB}+G_{SA}$$ (4)

In the Poisson-Boltzmann model (PB), the polar contribution is computed through the well-known PB equation. On the other hand, in the case of GB models, the polar solvation component (G_GB_) is calculated through Eq 5 proposed by Still et al [63]. Even though the PB model is considered as a more rigorous approach, GB models are less computationally-demanding and often give fairly satisfactory predictions [61, 64].

$$G_{GB}\left(X\right)=\frac{1}{2}\left(1\frac{1}{\varepsilon_{\omega }}\right)\overset{}{\underset{i,j\in X}{\sum }}q_{i}q_{i}{\left[r_{ij}^{2}+R_{i}R_{j}\exp\left(\frac{r_{ij}^{2}}{4R_{i}R_{j}}\right)\right]}^{\frac{1}{2}}$$ (5)

The term ε_w_ is the dielectric constant of the solvent (e.g. water). i and j represent the solute atoms, being r_ij_ the distance between them, q_i_ and q_j_, their partial charges, and R_i_ and R_j_, their effective Born radii.

The non-polar solvation contribution is calculated through Eq 6, where SA stands for the solvent-accessible surface area of the solute. Coefficients γ and β are empirical constants with values of 0.0072 kcal/mol and 0, respectively, for the GB models [65].

$$G_{SA}=\gamma SA+\beta$$ (6)

In this study, MM-GBSA free energy calculations were performed using MMPBSA.py module of AMBER12 [66]. The snapshots of the complex, the receptor and the ligand were extracted from a single desolvated trajectory. GB^OBC1^ model (igb = 2) with mbondi2 radii [67] was used for estimating ΔG_GB_. Snapshots belonging to the equilibrated trajectory were used for the calculation of effective binding free energy (ΔG_eff_), which comprises all the energy terms in the right-hand member of Eq 2 except for the entropy contribution. Additionally, the snapshots were considered as statistically independent from each other during ΔG_eff_ calculations. Conformational entropy associated with ligand binding was estimated by Normal-Mode Analysis (NMA) [68, 69]. For entropy calculations, seventy frames evenly extracted from the productive MD simulations were taken. Prior to normal-mode calculations the complex, the ligand and the receptor were subjected to 50000 cycles of EM using a distance-dependent dielectric constant of 4*r* (*r* being the distance between atom pairs) and a dmrs value of 10^–4^ kcal/(mol Å) as the convergence criterion for the root-mean squared gradient. Per-residue effective free energy decomposition (ΔG_res_) was carried out in order to determine the more important residues involved in FhCL3-ligand and FhCL3-substrate interactions [70]. Also, pair-wise effective free energy decomposition was performed for the FhCL3-substrate complex [70].

### Trajectories analysis and determination of protein-ligands interactions

The trajectories were analyzed with tools provided by GROMACS v4.6.3 package [52]. The Root Mean Square Deviation (RMSD) was calculated during the productive run with respect to both the starting and average structures. Visual Molecular Dynamics (VMD) v1.9.1 [71] was used to visualize trajectories and to convert the GROMACS MD trajectory format (xtc) into AMBER trajectory format (crd). Hydrogen bonds established between each ligand and FhCL3 were calculated employing a donor-acceptor distance cutoff ≤ 3.5 Å and a donor-acceptor-hydrogen angle cutoff ≤ 30 degrees during the equilibrated productive trajectory. PYMOL v1.6 [72] and LigPlot [73] were used for visualization, and Gnuplot v4.4 for graphic analysis of time profiles.

### Accessions Numbers

UniProtKB: Q9GRW6

Protein Data Bank (PDB): 2O6X, 2YJC, 1PPP, 1CVZ, 9PAP and 1ATK

## Results and Discussion

### Energetic characterization of the interaction interface of FhCL3 in complex with a peptidic substrate

A 3D-model of FhCL3 was generated based on a MSA of twelve papain-like proteases (S1 Fig) and using the crystal structure of proFhCL1 C25G (PDB: 2O6X) [14] as a template. The assessment methods employed here confirmed the high quality of the 3D-model of FhCL3 (S1 Table and S1–S4 Figs), thereby suggesting its suitability for further structure-based analyses.

**Fig 1:**
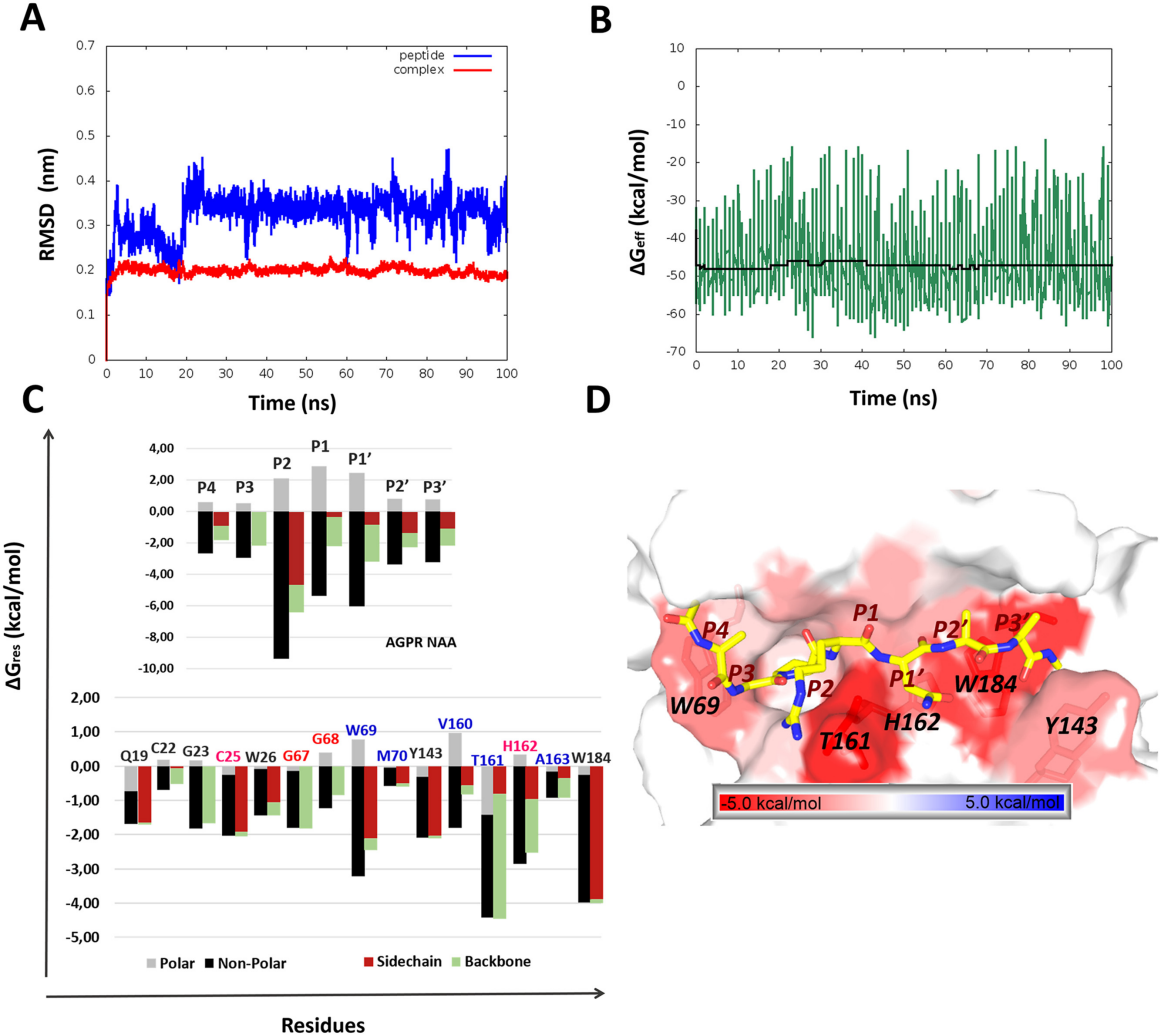
Stability assessment and per-residue free energy decomposition for the FhCL3-peptide complex. (A) RMSD values relative to the initial structure calculated for the backbone atoms of the peptide (blue) and the FhCL3-substrate complex (red)during the simulation time. (B) Effective binding free energy (green) and accumulated mean (black) values versus simulation time. (C) Per-residue free energy values for key residues of the FhCL3-peptide complex. Bars are split into backbone, side chain, polar and non-polar contributions. Residue names are colored according to the enzyme’s subsite location, i.e. S1 in pink, S2 in blue and S3 in red. (D) Structural representation of FhCL3 hot-spots according to per-residue energy contribution values onto the average structure of the complex.

MD simulations in combination with MM-GBSA per-residue and pair-wise free energy decomposition were performed for the FhCL3-peptide complex. Convergence and stability of the MD simulation was monitored through the inspection of structural and energetic properties. RMSD values showed different time evolution when calculated for the heavy atoms of the whole complex and for those of the peptide (Fig 1A). In this regard, the whole complex showed relatively stable RMSD values, whereas the peptide displayed structural fluctuations during the first 20 ns, indicating a delay on its stabilization into binding site. This difference is a consequence of the slight contribution of the peptide atoms to the global RMSD. Additionally, instantaneous ΔG_eff_ values were calculated (Fig 1B). It is noteworthy that the accumulated mean values of ΔG_eff_ reached relatively stable values during the MD simulation. Overall, these results suggest that 20 ns is a suitable equilibration time and, therefore, the last 80 ns were used to calculate mean ΔG_eff_ values.

Ten residues, i.e., Q19, G23, G25, W26, G67, W69, Y143, T161, H162 and W184, of FhCL3 largely contribute to the substrate binding (ΔG_res_≤-1.0 kcal/mol) according to the predictions of the per-residue free energy decomposition protocol (Fig 1C). Our results showed that most peptide-FhCL3 interactions are governed by non-polar contributions (i.e., mainly van der Wals interactions). It is worth noting that some residues widely conserved within the cathepsin L family, i.e., Q19, G23, C25, W26, G67, H162 and W184 (S1 Fig) are included within the previous list. Particularly, the catalytic residues C25 and H162 are hot-spots (residues whose side chain contribute in more than 1 kcal/mol to ΔG_eff_) which establish important pair-wise interactions with the peptide residues from the P2 to the P1’ site (Table 1). Q19 is another hot-spot with a large electrostatic per-residue contribution located within the oxyanion hole of the enzyme [74]. This residue strongly interacts with the P1’ site residue through the formation of a hydrogen bond (S5 Fig). Likewise, G67 was predicted as an important residue for anchoring the substrate through the formation of a hydrogen bond, in this case, with the residue at P2 (S5 Fig). Interestingly, equivalent interactions involving Q19 and G67 have been observed in the 3D structures of other papain-like proteases in complex with peptidomimetic compounds [75, 76]. Finally, the side chain of W184 and, to a less extent, that of W26 establish favorable van der Waals interactions with residues at P1’-P3’ and P2 sites, respectively (Table 1 and Fig 1C and 1D). Overall, our predictions are in agreement with the essential roles attributed to some of the previously-mentioned residues within the papain-like family.

**Table 1:** Pair-wise energy decomposition values for FhCL3-sustrate complex.^a^

Interacting residues		van der Waals	Electrostatic	Polar Solvation	Total
**A(P4)**	G68	-0.95±0.38	-0.98±0.56	0.71±0.28	-1.21±0.72
	W69	-1.81±0.66	-1.59±0.80	0.62±0.37	-2.78±1.10
**G(P3)**	G68	-0.57±0.30	-1.90±0.81	0.69±0.20	-1.79±0.89
	W69	-1.81±0.59	-1.67±0.49	0.33±0.24	-3.16±0.80
	V160	-1.01±0.41	0.26±1.00	-0.26±0.58	-1.01±1.22
**P(P2)**	C25	-0.99±0.43	-0.75±0.49	0.16±0.37	-1.59±0.75
	W26	-1.77±0.44	-1.65±0.73	1.45±0.39	-1.97±0.94
	G67	-0.57±0.39	-4.12±0.53	1.35±0.21	-3.34±0.69
	G68	-1.17±0.72	-1.34±0.98	-0.08±0.27	-2.58±1.24
	W69	-2.22±0.52	0.90±0.34	-0.37±0.20	-1.69±0.65
	M70	-0.87±0.25	0.13±0.14	-0.26±0.10	-1.00±0.30
	T161	-1.39±0.49	-1.92±0.53	0.34±0.15	-2.97±0.74
	H162	-1.87±0.35	-1.01±0.30	0.69±0.16	-2.19±0.49
	A123	-1.11±0.28	-0.27±0.09	-0.04±0.05	-1.41±0.30
**R(P1)**	G23	-1.19±0.60	-3.19±1.00	2.28±0.48	-2.10±1.28
	G67	-1.18±0.35	3.43±1.01	-3.52±0.96	-1.28±1.43
	T161	-1.61±1.07	-6.17±1.30	0.79±0.91	-6.99±1.91
	H162	-0.68±0.13	-4.18±0.63	3.25±0.51	-1.62±0.82
**N(P1’)**	Q19	-0.06±0.80	-4.24±1.45	0.10±0.55	-4.20±1.75
	G23	-0.93±0.26	-0.08±0.54	-0.18±0.25	-1.19±0.65
	C25	-1.20±0.26	-2.45±0.92	1.37±0.35	-2.29±1.02
	Y143	-0.36±1.36	-4.74±3.72	0.93±1.15	-4.16±4.12
	T161	-1.09±0.82	-3.05±1.86	-0.02±0.63	-4.16±2.13
	H162	-2.51±0.47	-0.02±0.63	0.40±0.41	-2.14±0.89
	W184	-1.47±0.56	-3.21±1.34	1.69±0.44	-2.99±1.52
**A(P2’)**	Q19	-0.91±0.33	0.26±0.34	-0.34±0.20	-0.99±0.51
	W184	-1.06±0.43	-0.44±0.49	-0.08±0.24	-1.58±0.70
**A(P3’)**	Y143	-0.91±0.64	-0.10±0.68	-0.01±0.37	-1.02±1.01
	W184	-2.31±0.80	-1.23±1.03	-0.28±0.47	-3.82±1.39

^a^ All energies are in kcal/mol

On the other hand, some non-conserved FhCL3 residues such as W69, Y143 and T161 have the largest per-residue free energy contributions to the complex formation (Fig 1C and 1D). W69 establishes strong van der Waals interactions with the substrate residues Ala(P4), Gly(P3) and Pro(P2) (Table 1). Interestingly, our predictions showed that the ring of Pro(P2) adopts a nearly-perpendicular conformation with respect to the indol group of W69 (Fig 1D), which precludes the stabilization through stacking interactions proposed before [21, 25]. Probably, a crucial role of Pro at the P2 site, given its bend-inducing capacity, is to promote the appropriate conformation of the substrate backbone within the enzyme binding site, especially that of the residue at P3, which strongly interacts with W69 (Table 1). In addition, the structural analysis showed the occurrence of close contacts between the backbone of residues at P4 and P3 sites with the indol ring of W69. Therefore, the substitution of the previous substrate residues by bulky amino acids could disrupt the interface complementarity thereby reducing the binding affinity as has been proven at least for the P3 site [25]. All these predictions clarify from an energetic point of view the experimental results that established the importance of W69 in determining the enzyme specificity for Gly and Pro residues at P3 and P2, respectively [21, 25, 27]. Of note, the MD simulation of the peptide-FhCL3 complex performed here also confirmed that the most representative rotameric configuration of W69 side chain in the bound state of the enzyme corresponds to that predicted by Corvo *et al* based on molecular modeling approaches [25]. This particular conformation partially occludes the S2 subsite and favors the interaction with Gly(P3) as predicted through our energetic analysis (Table 1) and also suggested before [25, 27]. In the case of Y143, it was predicted the formation of a hydrogen bond comprising its phenol group and the carbonylic oxygen of Asn(P1’) (S5 Fig), which, in addition to its van der Waals interaction with Ala(P3’), explains the large energy contribution of this residue (Table 1 and Fig 1D). In general, we believe that the interaction with Y143 might enhance the specificity of ligands toward FhCL3, given that this residue is not conserved within the papain-like family and also bears a hydroxyl group with the capacity of forming specific hydrogen bonds with the substrate.

Additionally, we obtained that the backbone of T161 has the largest energy contribution to the complex formation (Fig 1C and 1D), which mainly arises from the hydrogen bonds involving its carbonyl oxygen (O) and the amidic hydrogen atoms of residues at P1 and P1’ sites (S5 Fig). Note that although position 161 is not conserved throughout the papain-like family [21] (S1 Fig) and its energy contribution to the substrate binding is large, at least for the complex analyzed here, its nature seems to be irrelevant. The latter stems from the fact that its interactions with the peptide residues are mediated mostly by its backbone oxygen atom rather than by its side chain hydroxyl group, which extends away from the interface in disagreement with previous suggestions [21]. Remarkably, equivalent interactions have been observed between D158 of papain and the amidic hydrogen atoms at the P2 site of petidomimetic inhibitors (PDBs: 1PPP and 1CVZ) [75, 77], thereby reinforcing our previous predictions.

Finally, we predicted the low energy contribution of H63 to the substrate binding. Hence, this position is not likely to be involved in ligand binding. This result explains the little impact of the H63N mutation on the specificity substrate profiles of FhCL3 [25].

### Virtual screening

**Fig 2:**
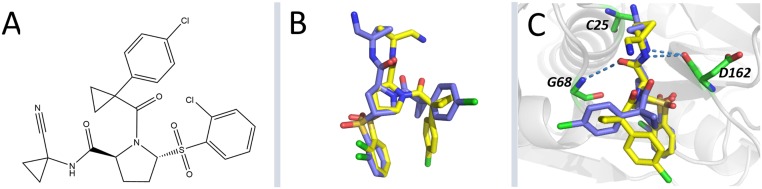
Nitrile re-docking into the substrate-binding site of HuCatL. (A) Nitrile chemical structure. (B) Superposition of nitrile best-re-docked pose (yellow) and crystal structure (blue) (RMSD = 3.4 Å). (C) Hydrogen bonds between the residues of HuCatL binding site and nitrile in both the crystal (blue) and the best re-docked (yellow) complex structures. Hydrogen bonds are shown as blue dotted lines, and HuCatL interacting residues (green) are depicted as sticks. Note that nitrile re-docking only takes into account its non-covalent interactions with the enzyme, therefore, it was treated as a non-covalent ligand.

The VS protocol used for the identification of potential ligands of FhCL3 was first validated through the non-covalent re-docking of nitrile within the binding site of HuCatL. This inhibitor has the sulfone chemical group (Fig 2A), which is common in many parasitic cysteine protease inhibitors [78, 79]. Irreversible inhibition mechanism occurs through covalent bond formation with the thiolate of the catalytic cysteine [80]. The RMSD for the heavy atoms of the re-docked pose having the highest S_vina_ value with respect to the experimental binding mode was only 3.4 Å (Fig 2B). Additionally, hydrogen bonds between nitrile and residues G68 and D162, were also reproduced in the predicted HuCatL-nitrile complex (Fig 2C). Note that only non-covalent interactions were taken into account in the re-docking simulation, therefore, the FhCL3-nitrile pre-complex rather than the actual covalent complex was modelled here. Overall, this procedure provided a reasonable prediction of nitrile experimental binding mode which, in turn, validates our docking protocol.

In order to reduce cross-inhibition between host and parasite targets, we took into account only the potentially-selective ligands for the parasitic enzyme. Through VS calculations, twelve compounds with relative binding free energy values lesser than -1.50 kcal/mol (ΔS_vina_<-1.50 kcal/mol) between FhCL3 and HuCatL complexes were identified (S2 Table). Moreover, according to the DrugMint scores, only five compounds constitute potential drug-like non-peptidic inhibitors (S2 Table). The names of these selective compounds and their S_vina_ values are listed in Table 2.

**Table 2:** Vina score, IUPAC name and MYBIDGE-HitFinder identifier for five selected compounds.

MYBRIDGE ID	S_vina_ (kcal/mol)	ΔS_vina_ (kcal/mol)	IUPAC name
**HTS11101**	-8.3	-2.4	4-(2,5-dimethyl-1H-pyrrol-1-yl)-N'-(5-methyl-3-phenyl-1,2-oxazole-4-carbonyl)benzohydrazide
**BTB03219**	-8.1	-1.6	1-N-[3,5-bis (trifluoromethyl) phenyl]-2-N-(1-ethynylcyclohexyl) benzene-1,2-dicarboxamide
**HTS12701**	-8.0	-1.8	2-{[4-benzyl-5-(pyridin-4-yl)-4H-1,2,4-triazol-3-yl]sulfanyl}2-1-(1,2,3,4-tetrahydroquinolin-1-yl)ethan-1-one
**RH01594**	-7.7	-2.6	(2E)-N'-{2-[(naphthalen-1-ylmethyl)sulfanyl]acetyl}-3-phenylprop-2-enehydrazide
**SPB07884**	-7.3	-1.6	4-[4-(benzyloxy)phenyl]-3-{[(4-methylphenyl)methyl]sulfanyl}-4,5-dihydro-1H-1,2,4-triazol-5-one

**Fig 3:**
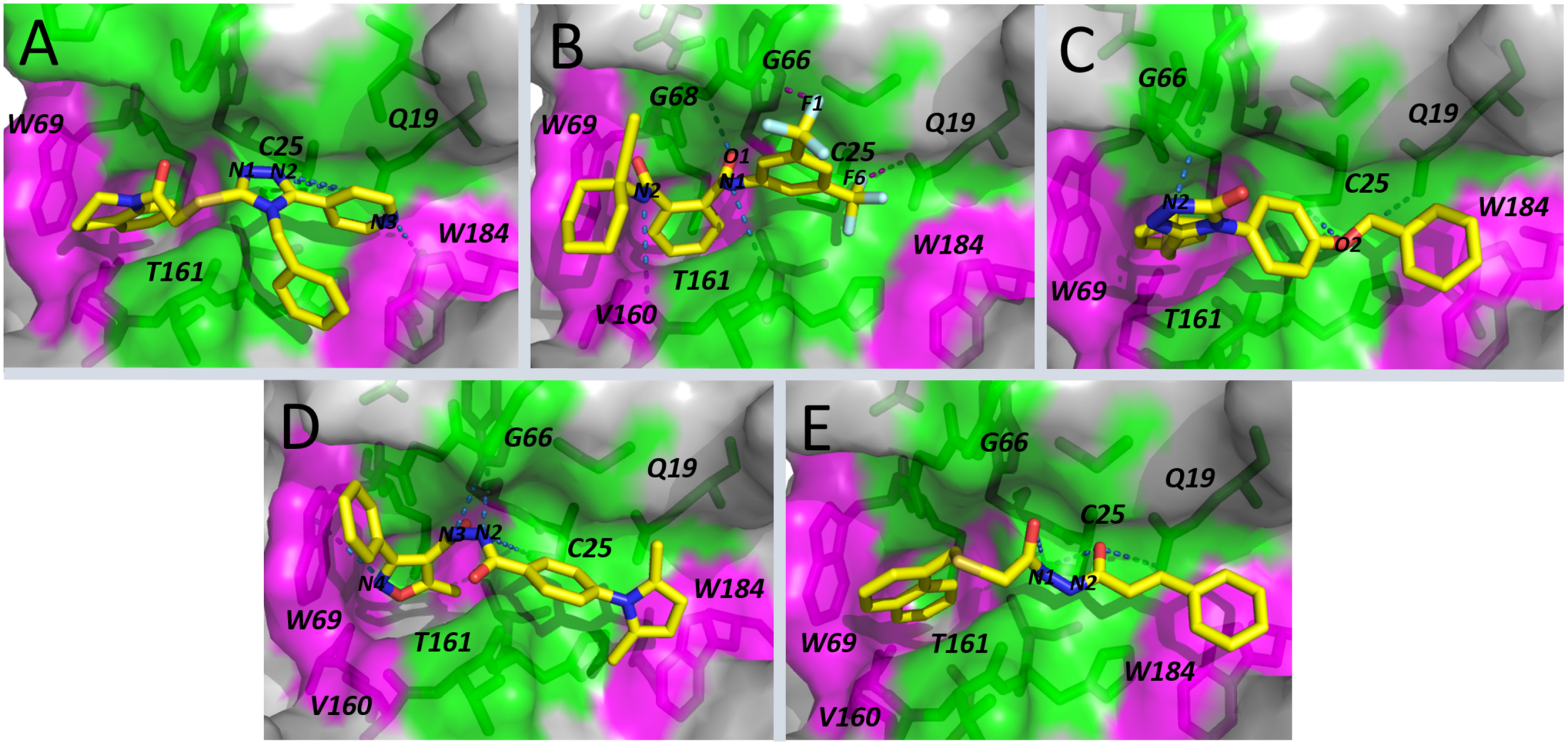
Selected docking poses for the best five hits. FhCL3 in complex with HTS12701 (A), BTB03219 (B), SPB07884 (C), HTS11101 (D) and RH01594 (E). FhCL3 interacting residues as well as ligand atoms involved in possible hydrogen (blue dashed lines) and halogen (purple dashed lines) bonds are labeled. Protein surface is colored according to its polar (green) and non-polar (magenta) properties.

Non-peptidic inhibitors are considered as the best strategy for *in vivo* inhibition in order to avoid degradation by proteases. In this sense, structure analysis suggests a common peptidomimetic scaffold among the selected compounds. Besides, they all possess a certain number of aromatic moieties, i.e., phenyl, naphtalene and bencyl groups, which increases their hydrophobicity. Those moieties could establish favorable hydrophobic interactions with the non-polar residues of FhCL3 binding site (Fig 3). Interestingly, several cysteine protease inhibitors reported so far bear aromatic functional groups in their structures [43, 81, 82]. On the other hand, the heterocyclic rings, i.e., triazole, pyrrol and isoxazole, present in some of the selected compounds, could establish polar interactions with various active site residues (Fig 3).

Some of the selected compounds, i.e., HTS12701 and RH0159, share a thiomethylene-ketone moiety (Fig 3A and 3E), which is present in various reversible inhibitors of cysteine proteases from *Plasmodium falciparum* and *Leishmania donovani* parasites [81]. The inhibitory mechanism of these compounds might involve the formation of transition-state-like hemithioacetal complexes with C25 at the protease active site [81]. On the other hand, the hydrazide moiety was observed in compounds HTS11101 and RH01594 (Fig 3D and 3E). This moiety is frequently present in inhibitors of other parasite cysteine proteases [43, 81]. Finally, in the case of BTB03219, it can be highlighted the presence of an aryl_CF3 substituent, which could increase the affinity for FhCL3 through halogen bond formation (Fig 3B), as has been reported for other cysteine proteases like HuCatL [44].

The VS protocol performed in this paper led to the identification of five compounds as possible inhibitors of FhCL3. However, the refinement of docking results is needed to more accurately predict the binding modes of the compounds to the enzyme, as well as the absolute and relative binding free energy values of the complexes. In this sense, the combination of conformational space-exploring techniques and end-point free energy calculation methods such as MD simulations and MM-GBSA, respectively, constitutes a useful approach to assess the stability of protein-ligand complexes [83–85].

### Binding free energy analysis

**Fig 4:**
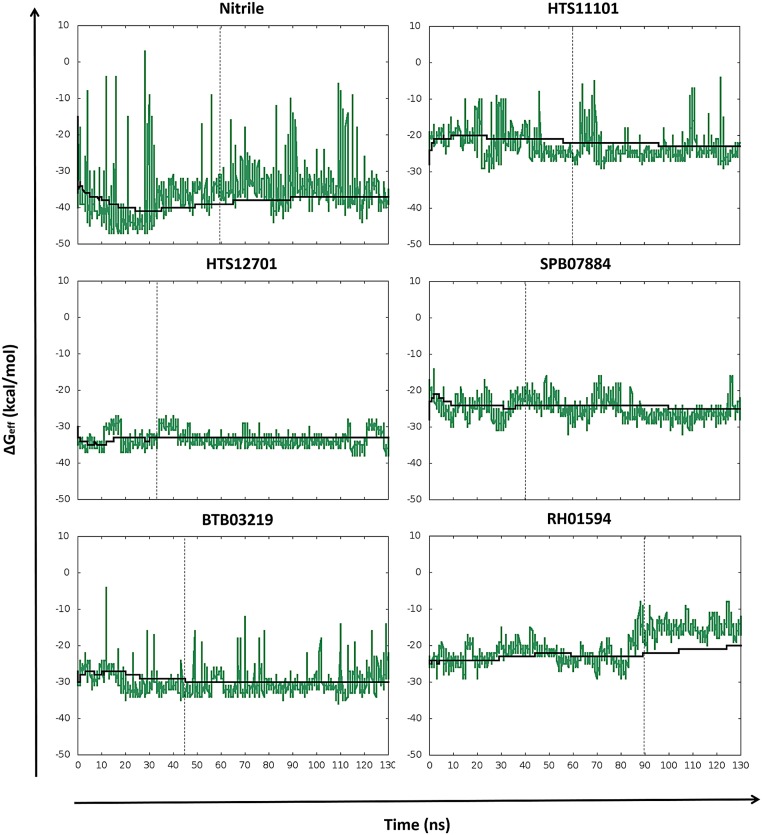
Time evolution of instantaneous ΔG_eff_ values for FhCL3 complexes. Effective binding free energies (green) are shown together with accumulated mean values (black). Dashed lines indicate the MD equilibration time of FhCL3 complexes. Every complex was labeled with the corresponding compound identifier.

Prior to free energy calculations, the stability and convergence of MD simulations were monitored through per-frame ΔG_eff_ time profiles. In this regard, fluctuations for the FhCL3-ligand complexes are shown together with accumulated mean values (Fig 4). ΔG_eff_ values are quite variable for each snapshot, but the accumulated mean values become stable in most of cases. Besides, significant differences in the stabilization time for every complex are clearly observed (Fig 4). The latter may result from the fact that for some FhCL3-ligand complexes the initial structures predicted by docking were farther from their MD average structures than for others (S6 Fig). Accordingly, the subsequent ΔG_bind_ calculations and the analyses of binding determinants and hydrogen bond formation are based on snapshots collected after the equilibration time of productive MD simulations.

To better understand the main energy components contributing to the formation of the different FhCL3-ligand complexes, we analyzed the MM-GBSA free energy components, i.e., van der Waals, electrostatic, polar solvation, non-polar solvation and entropy (TΔS) contributions (Table 3). The results indicate that non-polar contributions (ΔE_vdw_+ΔG_SA_) dominated the binding process, because the complex formation reduces the Solvent Accessible Surface Area (SASA) and the enzyme binding site has hydrophobic-interacting residues, i.e., W69, V160, V209 and W184, which establish favorable van der Waals interactions with the different ligands. Conversely, the polar (ΔE_elec_+ΔG_GB_) and entropy terms have unfavorable contributions in all cases.

**Table 3:** Binding free energies and its components for the studied systems.^a^

Systems	Nitrile	HTS12701	BTB02319	HTS11101	SPB07884	RH01594
**ΔE**_**vdw**_	-46.96±0.12 ^b^	-47.30±0.03	-41.70±0.08	-35.14±0.07	-37.95±0.05	-27.80±0.04
**ΔE**_**elec**_	-10.94±0.1	-9.89±0.03	-9.81±0.06	-15.73±0.036	-3.84±0.03	-13.81±0.07
**ΔE**_**MM**_	-57.90±0.16	-57.19±0.04	-51.51±0.1	-50.87±0.08	-41.79±0.06	-41.60±0.09
**ΔG**_**GB**_	28.30±0.08	28.19±0.02	25.67±0.05	30.86±0.02	20.54±0.03	28.99±0.06
**ΔG**_**SA**_	-5.60±0.003	-4.89±0.002	-4.98±0.004	-4.33±0.003	-4.38±0.004	-3.18±0.005
**ΔG**_**sol**_	22.70±0.07	23.30±0.02	20.69±0.05	26.53±0.02	16.16±0.03	25.81±0.06
**ΔG**_**eff**_ ^**c**^	-35.20±0.13	-33.89±0.03	-30.82±0.08	-24.33±0.07	-25.63±0.03	-15.79±0.04
**TΔS**	-24.64±0.50	-23.18±0.50	-22.66±0.42	-22.02±0.63	-22.75±0.43	-19.11±0.56
**ΔG**_**bind**_ ^**d**^	-10.56±0.52	-10.71±0.50	-8.16±0.43	-2.31±0.63	-2,88±0.43	3.32±0.56
**K**_**i**_ ^**e**^	(1.80±1.58)∙10^–8^	(1.40±1.18)∙10^–8^	(1.04±0.75)∙10^–6^	(2.02±2.15)∙10^–2^	(7.72±5.61)∙10^–3^	(2.67±2.57)∙10^2^

^a^ All energies are in kcal/mol

^b^ Standard Errors of mean

^c^ Effective free energy Δ*G*_*eff*_ = Δ*E*_*MM*_+Δ*G*_*sol*_

^d^ Binding free energy Δ*G*_*bind*_ = Δ*G*_*eff*_-TΔ*S*

^e^ Inhibition constant obtained from Δ*G*_*bind*_ = -*RT*ln(*K*_*i*_), where the temperature T is determined as 300K and values are given in mol/L.

MM-GBSA results show that HTS12701 is the compound with the best ΔG_bind_ value (-10.71 kcal/mol), followed by BTB03219 (-8.16 kcal/mol), both of them similar to the positive control (nitrile, -10.55 kcal/mol). Furthermore, K_i_ values calculated from theoretical ΔG_bind_ values suggest that HTS12701 and BTB03219 bind the enzyme in the sub-micromolar and micromolar concentration ranges (Table 3 **)**, respectively, the former being predicted as a tight-binding inhibitor. The theoretical K_i_ values for the rest of the ligands predict their low affinity interactions with the enzyme. Interestingly, the comparison between both the average structure of the productive MD simulation and initial docking pose shows that compounds like HTS12701 and BTB03219 keep bound to FhCL3 active site through specific interactions like nitrile (S6A–S6C Fig). Particularly, the former compound adopts a conformation during the MD simulation which places the thiomethylene ketone moiety closer to C25, in agreement with the proposed binding mode of this compound series [81]. On the other hand, the remaining compounds, i.e., SPB07884, HTS11101 and RH01594 (S6D–S6F Fig) do not form stable complexes during simulation time, which explains their more unfavorable ΔG_bind_ values.

Finally, by comparing the MM-GBSA and S_vina_ values we can observe changes in the ranking list of the selected compounds. However, the energy values lie within the similar ranges of free energy values (from -11 kcal/mol to -8 kcal/mol) for the best hits (compare Tables 2 and 3). As MM-GBSA is considered to be a more accurate method for estimating relative affinities [61, 64, 83], its predictions were taken as the final criterion for hit ranking.

### Per-residue free-energy decomposition—Insights into FhCL3-ligand interactions

**Fig 5:**
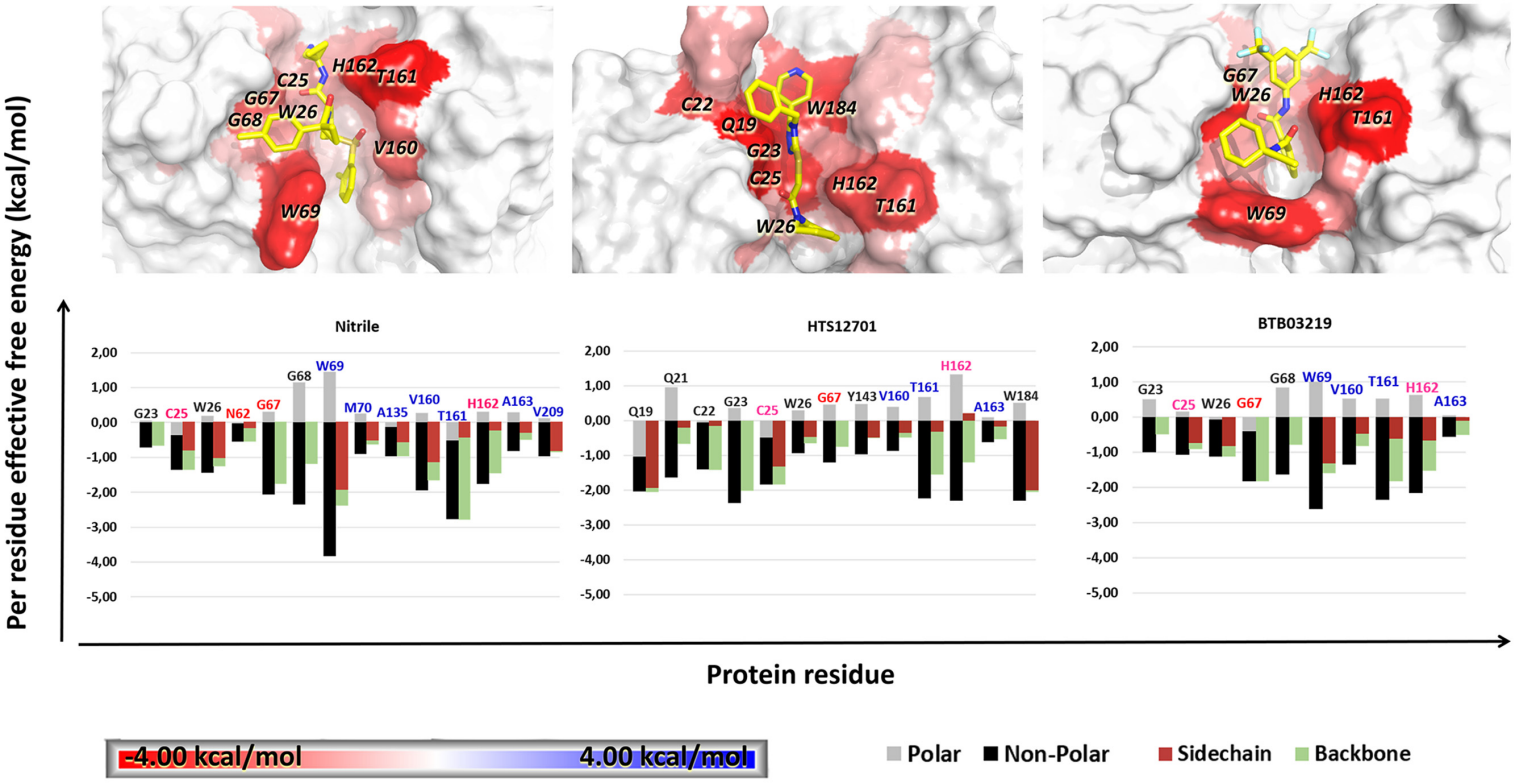
Per-residue free energy decomposition for FhCL3 complexes. Bar graphs show the side chain, backbone, polar and non-polar contributions for each residue. Residue names are colored according to their location within S1 (pink), S2 (blue) and S3 (red) subsites. A structural representation of each complex interface is depicted as well. Interacting residues are colored according to energy value as shown in color scale. Hot/warm-spots are labeled in each case.

In order to get insights into FhCL3-ligand interactions, the MM-GBSA approach was employed to decompose the binding effective free energy of the high-affinity complexes into per-residue contributions (Fig 5). This allowed us to identify the residues at the enzyme active site with significant energy contributions to the ligand binding. In general, we observed that some of these residues, e.g. G67, V160 and T161 lie within the S2 and S3 subsites, indicating substrate-like binding modes of the ligands to FhCL3. The subsequent decomposition of ΔG_res_ into backbone and side chain energy contribution led to the identification of six critical (warm/hot-spot) residues, i.e., Q19, C25, W69, V160, W184 and V209, whose respective side chain energy contributions were larger than those of the backbone. The latter suggests the essential role of these specific residues in the formation of some FhCL3-ligand complexes (Fig 5). It is also worth noting the prevalence of per-residue non-polar energy contribution in all systems, in agreement with ΔG_eff_ decomposition results shown in the previous section. Accordingly, most of the previously-mentioned residues are hydrophobic and, thus, can establish strong van der Waals interactions with compounds containing aromatic groups (Fig 5).

**Fig 6:**
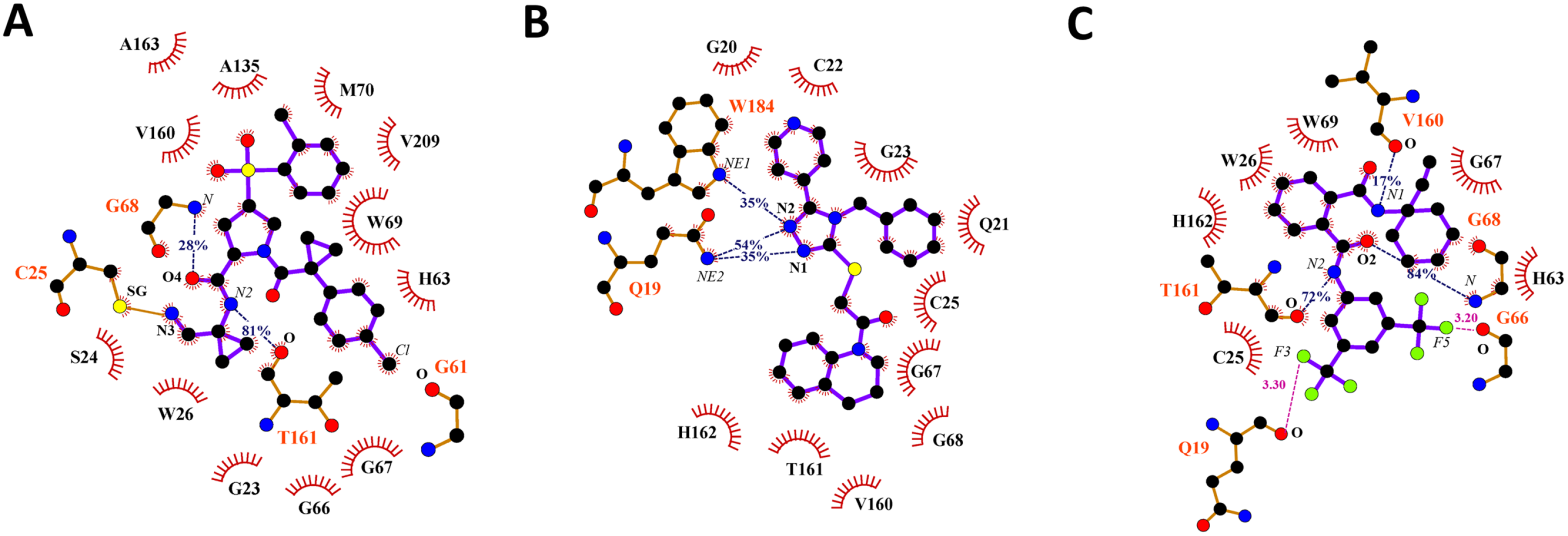
Diagrams of protein-ligand interactions. FhCL3 residues interacting with Nitrile (A), HTS12701 (B), BTB03219 (C). Ligands (violet) and protein residues involved in polar interactions (brown) are depicted in ball and stick representation. Hydrogen (blue dashed lines) and halogen (purple dashed lines) bonds are shown together with their occupancy percent and distance values, respectively. Hydrogen donor and acceptor labels are shown in italic and bold styles, respectively. Residues establishing non-polar contacts are depicted as red semicircles.

For the FhCL3-nitrile complex, four energetically-relevant residues, i.e., G67, W69, V160, and T161 (Fig 5), were identified. W69 and V160 are likely to interact with hydrophobic moieties (Fig 6A), while T161 forms a stable hydrogen bond with the N2 atom of nitrile, equivalent to that described before for the FhCL3-peptide complex (Fig 6A). Overall, these results are consistent with a previous work which state that nitrile non-covalent interactions comprise the enzyme active site and, especially, the specificity substrate subsites (S2 and S3) [44].

The complexes of FhCL3 with the best hits, i.e., BTB03219 and HTS12701, and nitrile share a group of common energetically-relevant residues, i.e., G23, C25, W26, G67, V160, T161and H162 (Fig 6). However, some other residues show significant differential energy contributions among the three complexes. For example, W69, which largely contributes to the formation of FhCL3-nitrile and FhCL3-BTB03219 complexes, seems to be irrelevant for HTS12701 binding to the enzyme. A similar behavior was observed for G68. Conversely, HTS12701 establishes strong interactions with Q19 and W184, residues belonging to S1-S1’ subsites, not observed in the other two complexes. Both residues are conserved throughout the cathepsin L family, which suggests their essential role in substrate binding, as observed for the FhCL3-peptide complex analyzed before. Therefore, HTS12702 may display low selectivity toward the proteases of this family. On the other hand, BTB03219 preferentially interacts with residues of the S2–S3 subsites, which are believed to control the enzyme specificity. Hence, though less potent than HTS12702, it is likely to be a more selective inhibitor. Note, however, that both compounds are predicted to display a roughly equivalent specificity for FhCL3 with respect to HuCatL, according to their respective ΔS_vina_ values (S2 Table).

Further insight into the structural determinants for the interaction of nitrile, BTB03219 and HTS12701 with FhCL3 was obtained through the hydrogen bond and hydrophobic contact analysis at the interfaces of these complexes. For example, W69 establishes hydrophobic interactions with aromatic rings of both nitrile and BTB03219, which is consistent with the large van der Waals energy contribution to the ΔG_res_ values of this residue (Fig 6). Another residue showing favorable hydrophobic interactions with the three compounds is V160, whose side chain interacts with the ligand hydrophobic moieties lying within the S2 subsite. On the other hand, the carbonyl oxygen atom (O) of T161 is involved in hydrogen bond formation with both BTB03219 and nitrile, which suggests the importance of this position in accommodating hydrogen donor groups within the S2 subsites. Interestingly, in this particular position the nature of the residue is irrelevant for protein-ligand interactions, as was also obtained for the FhCL3-peptide complex. Furthermore, Q19 at the S1’ subsite forms two alternative hydrogen bonds with acceptor nitrogen atoms of HTS12701. Unlike the previous case, the nature of this residue is important, since the hydrogen bond involves the NE2 atom of its side chain. In fact, Q19 is part of the oxyanion hole of cysteine proteases and stabilizes the tetrahedral intermediate of protease-substrate complexes [74]. Finally, the carbonyl oxygen atoms of Q19 and G66 may form halogen bonds with the fluorine atoms of BTB03219 (Fig 6), thereby contributing to the affinity of this particular compound for FhCL3. Interestingly, halogen bonds have been observed in the crystal structure of HuCatL in complex with nitrile and are believed to contribute to the binding process [44]. Similar analyses were carried out for the compounds with less favorable ΔG_bind_ values (S7 and S8 Figs).

A close inspection of the representative structures of the FhCL3-ligand complexes also revealed that the side chain of W69 can adopt different rotameric conformations depending on the nature of the ligand bound to the enzyme (Fig 5 and S7 Fig). Specifically, we observed that in the FhCL3-nitrile complex the side chain of W69 has a coaxial orientation with respect to the binding site, while in the other complexes it has a perpendicular conformation that partially occludes the S2 subsite, as obtained for the FhCL3-peptide complex analyzed before. It is worth saying that even though both rotameric conformations have been proposed before [25], this is the first time that their occurrence is predicted through MD simulations of FhCL3 in complex with different ligands. Therefore, our results reinforce the importance of W69 side chain rotation for the accommodation of ligands with different shapes within the enzyme binding site, as suggested before [21, 25].

Overall, we proposed some crucial interaction patterns between the selected compounds and FhCL3. Finally, as expected, the best hits interact with residues previously characterized for the FhCL3-peptide complex, which indicates their substrate-like binding mode. The list of most favorable residues (C25, W26, G67, V160, T161 and H162) for ligand interaction is roughly similar for most of the compounds analyzed here. Remarkably, there are some distinctive residues of FhCL3 mediating the interactions with the ligands through its side chains, i.e., W69 and V160, which have been identified as substrate-specificity determinants [21].

### Conclusions

In the present study, a computational protocol consisting of VS, MD simulations, and binding free energy calculations was used to search for novel and selective inhibitors against FhCL3. Additionally, the results obtained here enhanced our understanding of the binding determinants of this protease with peptidic substrates and organic ligands. Free energy calculation through a more accurate method, i.e., MM-GBSA, proved to be a useful post-docking refinement tool, since a new ranking list different from that of VS, was finally obtained. The further decomposition of the overall binding free energies into individual energy terms indicated that the van der Waals interactions are the dominant force for substrate/ligands binding. Moreover, the decomposition of the binding free energy into per-residue contributions showed that the non-polar side chain of residue W69 establishes critical van der Waals interactions with the substrate and some ligands. This agrees with a previous work that highlights the importance of this residue for FhCL3 specificity [25, 27]. Interestingly, we also observed that the side chain of this residue may adopt different conformations to accommodate different ligand groups within enzyme binding site. The previous results suggest that a flexible docking protocol allowing the rotation of the side chain of W69 would lead to the identification of more diverse scaffolds of FhCL3 ligands. Roughly six different residues, i.e., C25, W26, G67, V160, T161 and H162, were predicted as energetically-important for ligand and/or substrate anchoring inside the FhCL3 active site via hydrophobic and hydrogen bond interactions in almost all complexes. However, the nature of T161, one of the residues with very large energy contribution, seems to be irrelevant, since its main interactions involved the backbone oxygen atom, suggesting that the variation of this residue within the S2 subsites of papain-like proteases may not necessarily affect the substrate binding. Overall, we proposed HTS12701 and BTB03219 as promising lead compounds that could be FhCL3 inhibitors. We expect that the structural insights obtained in this study will facilitate the design of novel inhibitors against FhCL3.

## Supporting Information

S1 Fig Multiple sequence alignment of papain-like cysteine protease superfamily. (A) Multiple sequence alignment of Papain, Zingipain, FgCL1, FgCL2 (*F*. *gigantia* cathepsins L), FhCL1, FhCL2, FhCL3 (*F*.*hepatica* cathepsins L), SmCL1, SmCL3 (*Schistosoma mansoni* cathepsins L), HuCatL1, HuCatK and bovine cathepsin L1. Secondary structure information corresponds to the FhCL1 crystal structure (PDB: 2O6X). “aA” and “bB” letters represent alpha-helices and beta-sheets, respectively, while the dots (.) stand for loops. The catalytic residues are marked with a square. Finally, the red arrow indicates the starting point of pro-proteases (inactive form) and green arrow, that of the mature active enzymes. Residues are colored according to ClustalX color scheme. (B) Structural superposition of FhCL1 crystal (PDB: 2O6X) (dark gray) and papain structure (PDB: 9PAP) (light gray). Secondary structure is represented as tubes and colored according to structural information given in the previous alignment analysis.
(TIF)

S2 Fig Comparison of HuCatK and FhCL1 cavities. Superposition of HuCatK in complex with E64 inhibitor (PDB: 1ATK) (green) and proFhCL1 C25G (PDB: 2O6X) (violet) (RMSD = 0.53 Å). Cavities calculated by CASTp (grey surface) have similar values of surface area in both cases (20.33 and 19.40 Å^2^ respectively). E64 is shown as ball and sticks.
(PNG)

S3 Fig Superposition of FhCL3 3D models. (A) The RMSD computed for the best 16 models with respect to the model with lowest DOPE value. SW nomenclature correspond to the model calculated with the SwissModel server. (B) Three-dimensional structural aligment of the 16 models.
(TIF)

S4 Fig Quality factors of the selected FhCL3 model. (A) Prosa Z-score. (B) Ramachandran plots showing the most-favorable zones and disallowed regions. (C) Normalized QMEAN plot shows the standard deviation. (D) Density plot for QMEAN.
(TIF)

S5 Fig Scheme of hydrogen bonds formed between FhCL3 and peptide substrate. FhL3-peptide snapshot taken from the most representative conformation of MD simulations. Residues involved in hydrogen bond formation (green) and substrate (yellow) are in stick representation. Hydrogen bonds (blue) are showed with occupancy percentage. Donors and acceptors are labeled with italic and bold letter, respectively.
(TIF)

S6 Fig Superposition of docking poses and MD average structures of the studied complexes. FhCL3-Nitrile (A), FhCL3-HTS12701 (B), FhCL3-BTB03219 (C), FhCL3-SPB07884 (D), FhCL3-HTS11101 (E) and FhCL3-RH01594 (F). Protein surface is colored according to the hydrophobic (magenta) and hydrophilic (green) properties of the residues.
(TIF)

S7 Fig Per-residue free energy decomposition for FhCL3 complexes with low affinity. Bar graphs show the side chain, backbone, polar and non-polar contributions for each residue. Residue names are colored according to their location within S1 (pink), S2 (blue) and S3 (red) subsites. A structural representation of each complex interface is depicted as well. Interacting residues are colored according to energy value as shown in color scale. Hot/warm-spots are labeled in each case.
(TIF)

S8 Fig Diagrams of protein-ligand interactions. FhCL3 residues interacting with SPB07884 (A), HTS11101 (B), RH01594 (C). Ligands (violet) and protein residues involved in polar interactions (brown) are depicted in ball and stick representation. Hydrogen bonds (blue dashed lines) are shown together with their occupancy percent. Hydrogen donor and acceptor labels are shown in italic and bold styles, respectively. Residues establishing non-polar contacts are depicted as red semicircles.
(TIF)

S1 Table Assessment values with different quality parameters for FhCL3 models. ^**a**^ Selected model.
(DOCX)

S2 Table DrugMint results for twelve compounds selected from VS. (DOCX)
