# Correction: Insights into the Interactions of *Fasciola hepatica* Cathepsin L3 with a Substrate and Potential Novel Inhibitors through *In Silico* Approaches

**DOI:** 10.1371/journal.pntd.0003856

**Published:** 2015-06-15

**Authors:** The PLOS Neglected Tropical Diseases Staff

There is in an error in the citation. The name of the fourth author, Rosemberg de Oliveira Soares, appears incorrectly. Please see the corrected citation here: Hernández Alvarez L, Naranjo Feliciano D, Hernández González JE, Soares RO, Barreto Gomes DE, Pascutti PG (2015) Insights into the Interactions of *Fasciola hepatica* Cathepsin L3 with a Substrate and Potential Novel Inhibitors through *In Silico* Approaches. PLoS Negl Trop Dis 9(5): e0003759. doi:10.1371/journal.pntd.0003759
