# Supplementary figures and images for: Insights into the Interactions of Fasciola hepatica Cathepsin L3 with a Substrate and Potential Novel Inhibitors through In Silico Approaches
Source: PLoS Negl Trop Dis. 2015 May 15;9(5):e0003759. doi: 10.1371/journal.pntd.0003759 (PMC4433193; doi:10.1371/journal.pntd.0003759)

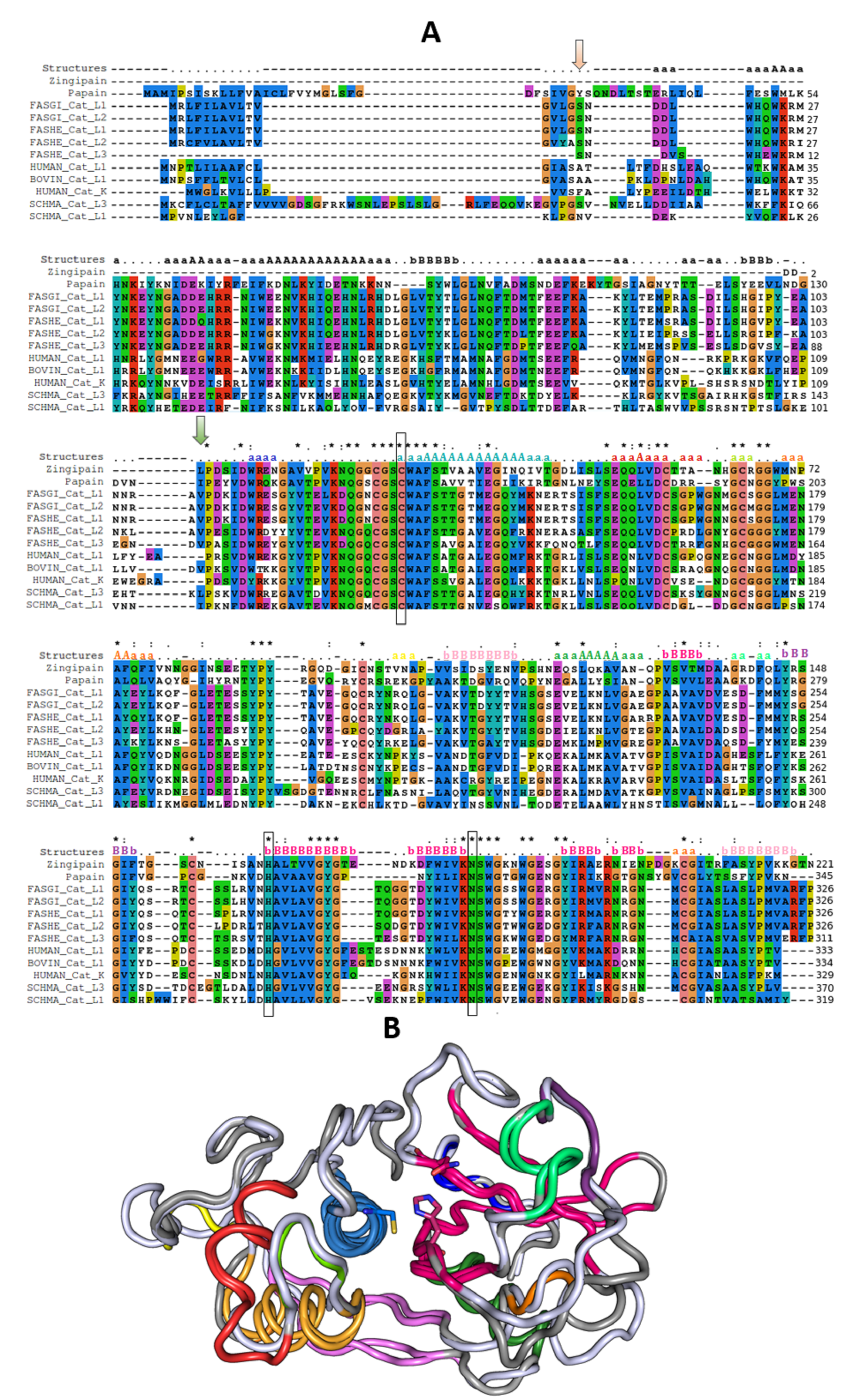

Supplement: S1 Fig — (A) Multiple sequence alignment of Papain, Zingipain, FgCL1, FgCL2 (F. gigantia cathepsins L), FhCL1, FhCL2, FhCL3 (F.hepatica cathepsins L), SmCL1, SmCL3 (Schistosoma mansoni cathepsins L), HuCatL1, HuCatK and bovine cathepsin L1. Secondary structure information corresponds to the FhCL1 crystal structure (PDB: 2O6X). “aA” and “bB” letters represent alpha-helices and beta-sheets, respectively, while the dots (.) stand for loops. The catalytic residues are marked with a square. Finally, the red arrow indicates the starting point of pro-proteases (inactive form) and green arrow, that of the mature active enzymes. Residues are colored according to ClustalX color scheme. (B) Structural superposition of FhCL1 crystal (PDB: 2O6X) (dark gray) and papain structure (PDB: 9PAP) (light gray). Secondary structure is represented as tubes and colored according to structural information given in the previous alignment analysis. (TIF) [file pntd.0003759.s001.tif]

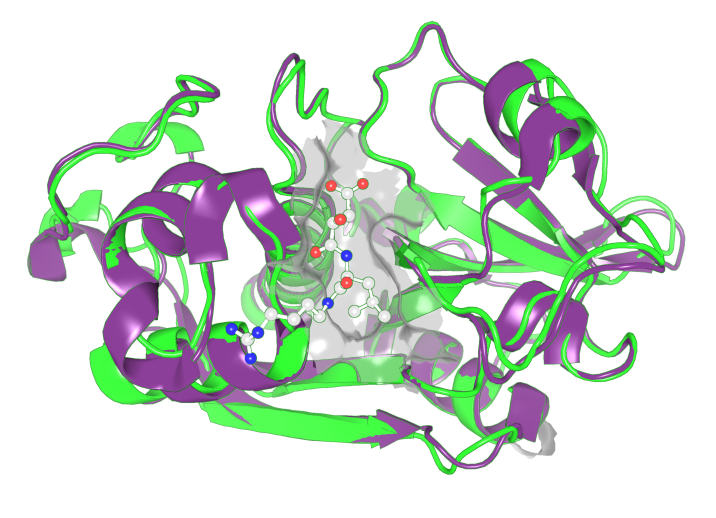

Supplement: S2 Fig — Superposition of HuCatK in complex with E64 inhibitor (PDB: 1ATK) (green) and proFhCL1 C25G (PDB: 2O6X) (violet) (RMSD = 0.53 Å). Cavities calculated by CASTp (grey surface) have similar values of surface area in both cases (20.33 and 19.40 Å2 respectively). E64 is shown as ball and sticks. (PNG) [file pntd.0003759.s002.png]

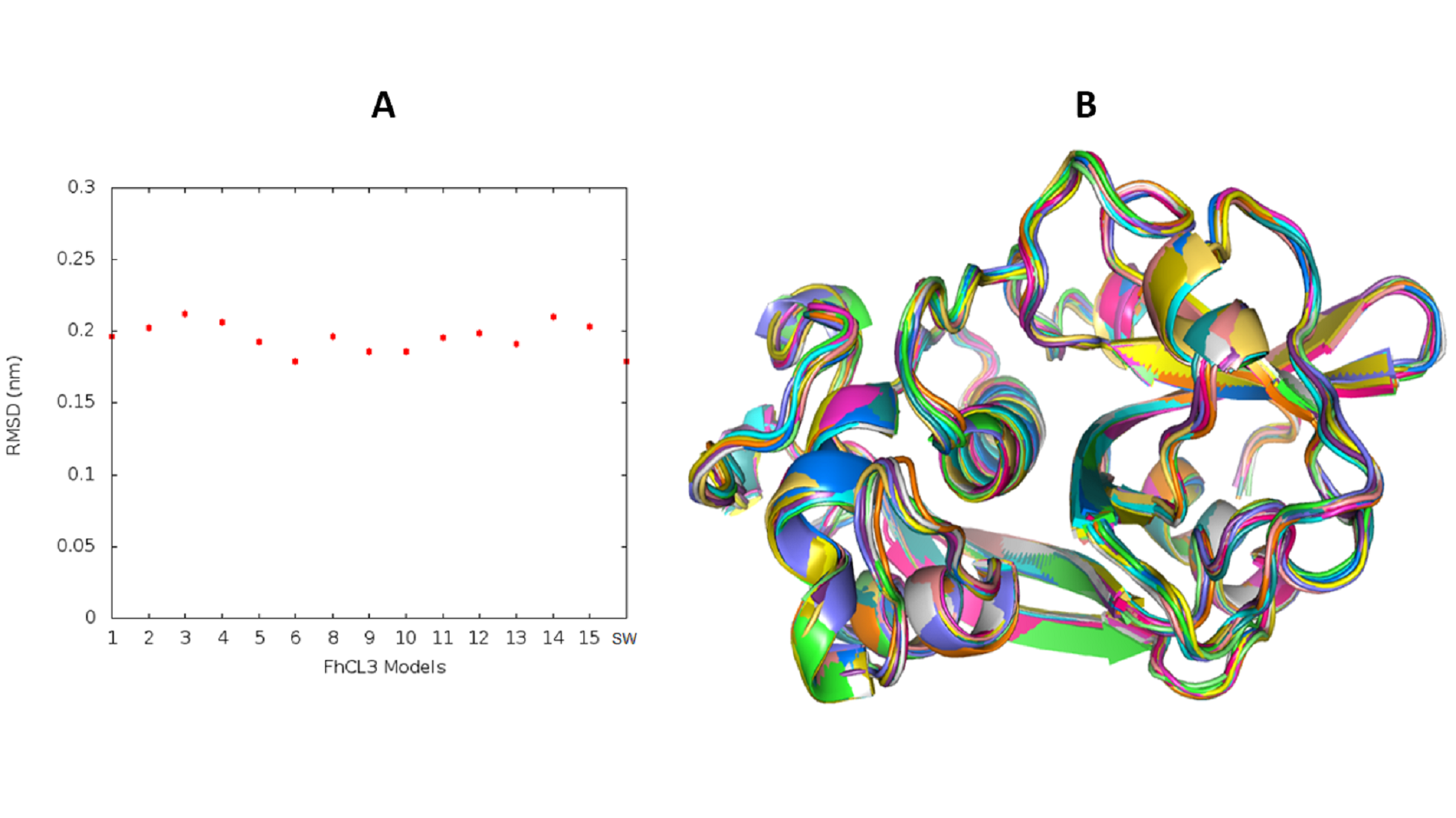

Supplement: S3 Fig — (A) The RMSD computed for the best 16 models with respect to the model with lowest DOPE value. SW nomenclature correspond to the model calculated with the SwissModel server. (B) Three-dimensional structural aligment of the 16 models. (TIF) [file pntd.0003759.s003.tif]

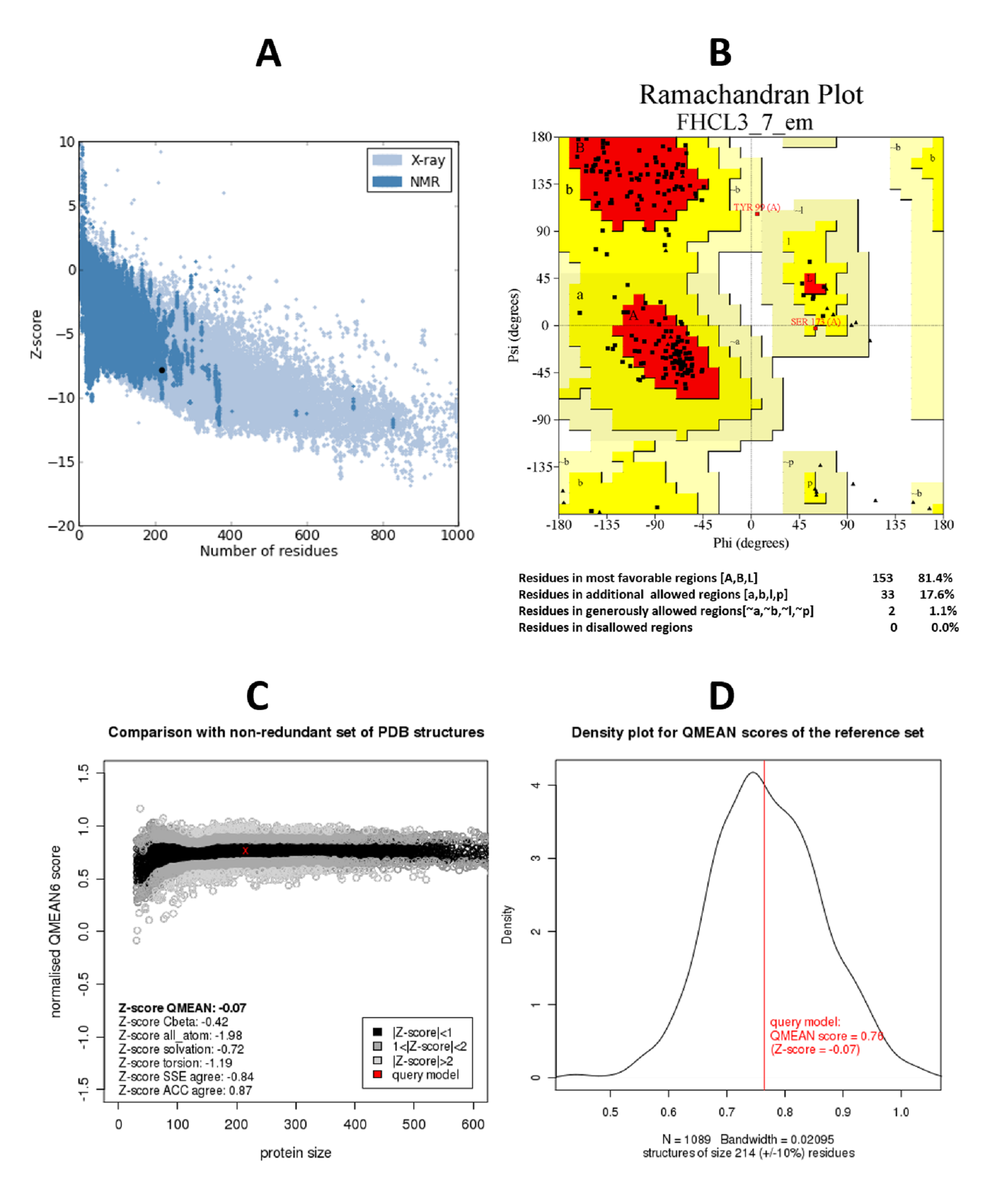

Supplement: S4 Fig — (A) Prosa Z-score. (B) Ramachandran plots showing the most-favorable zones and disallowed regions. (C) Normalized QMEAN plot shows the standard deviation. (D) Density plot for QMEAN. (TIF) [file pntd.0003759.s004.tif]

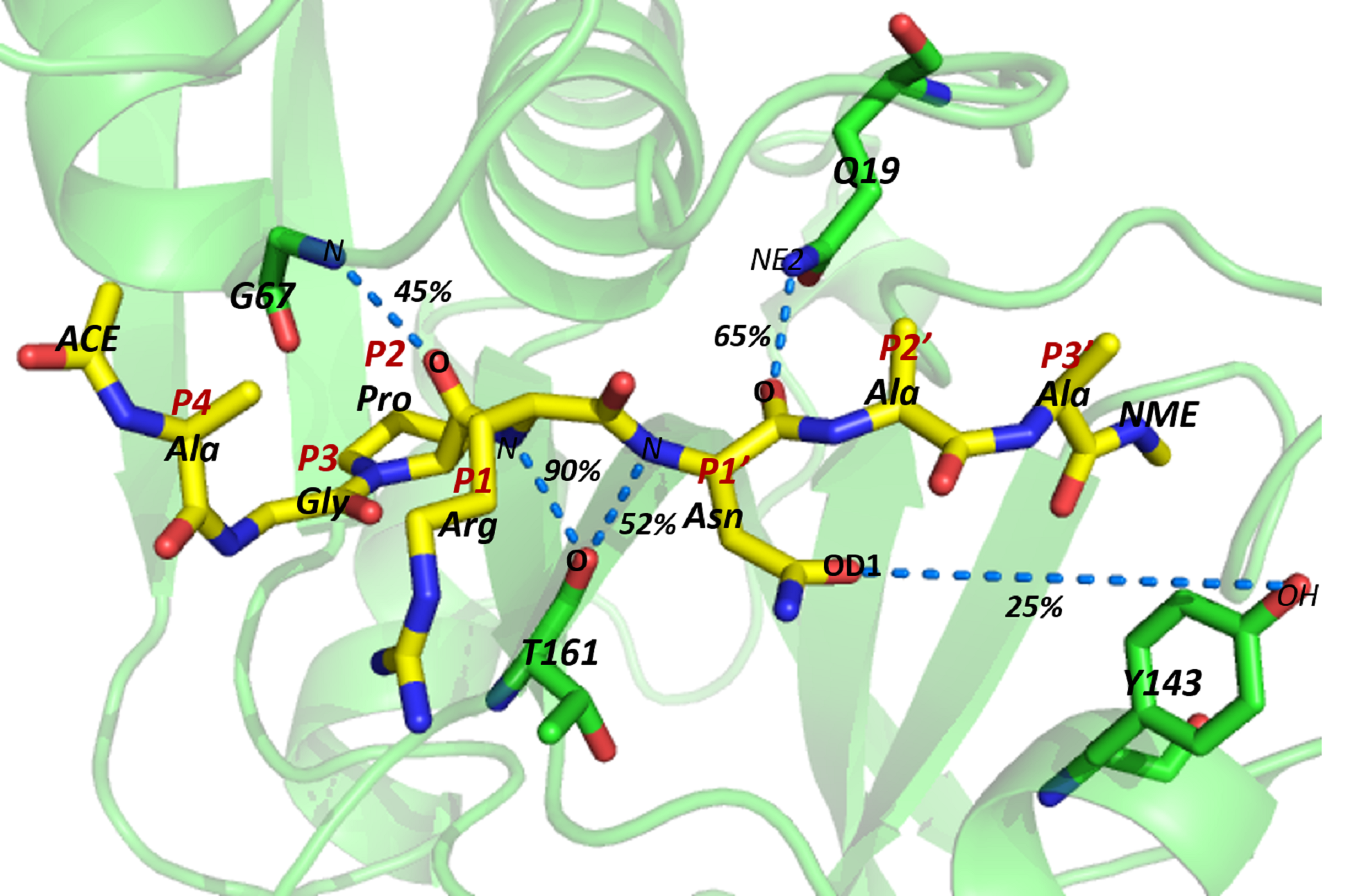

Supplement: S5 Fig — FhL3-peptide snapshot taken from the most representative conformation of MD simulations. Residues involved in hydrogen bond formation (green) and substrate (yellow) are in stick representation. Hydrogen bonds (blue) are showed with occupancy percentage. Donors and acceptors are labeled with italic and bold letter, respectively. (TIF) [file pntd.0003759.s005.tif]

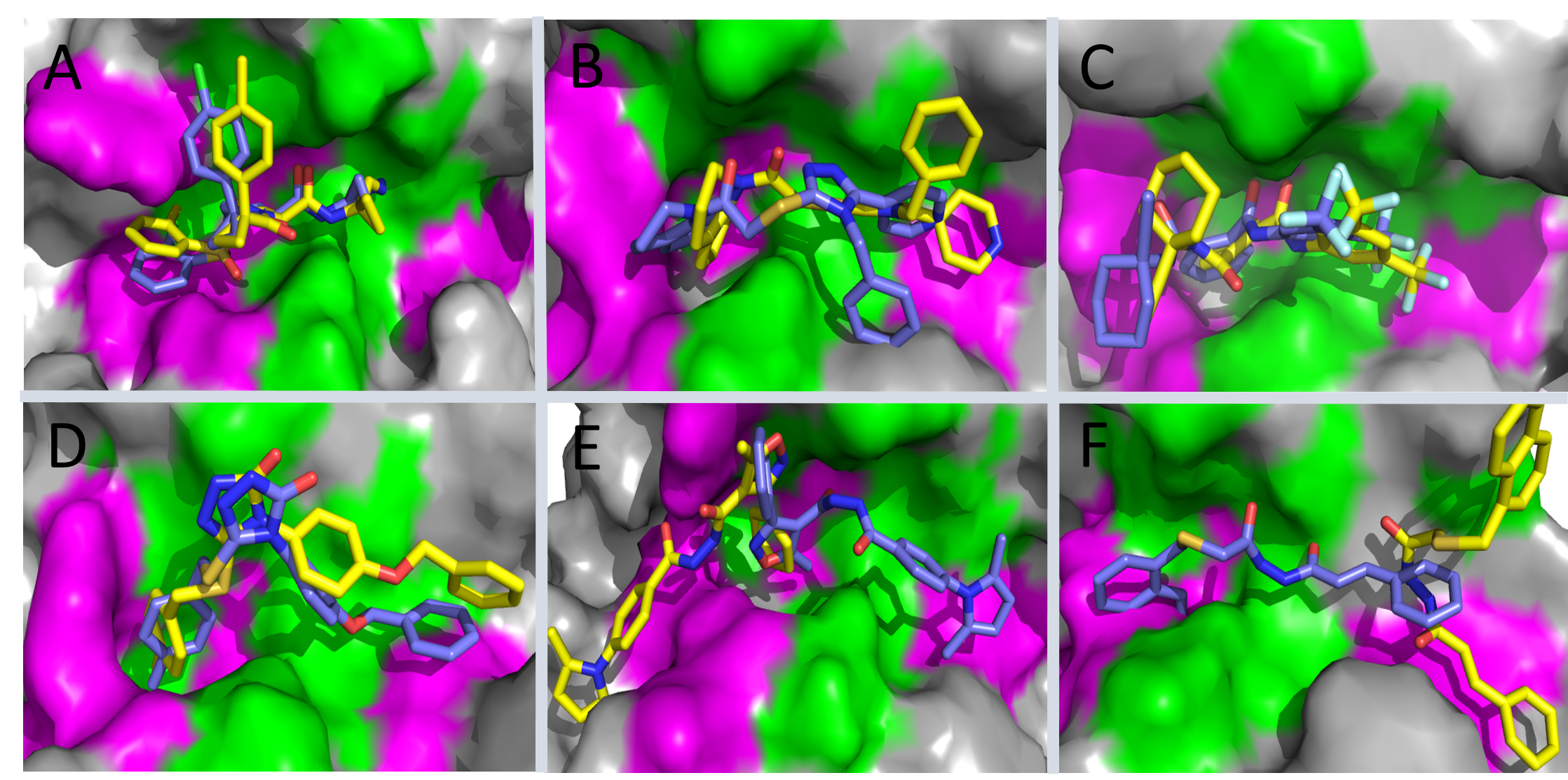

Supplement: S6 Fig — FhCL3-Nitrile (A), FhCL3-HTS12701 (B), FhCL3-BTB03219 (C), FhCL3-SPB07884 (D), FhCL3-HTS11101 (E) and FhCL3-RH01594 (F). Protein surface is colored according to the hydrophobic (magenta) and hydrophilic (green) properties of the residues. (TIF) [file pntd.0003759.s006.tif]

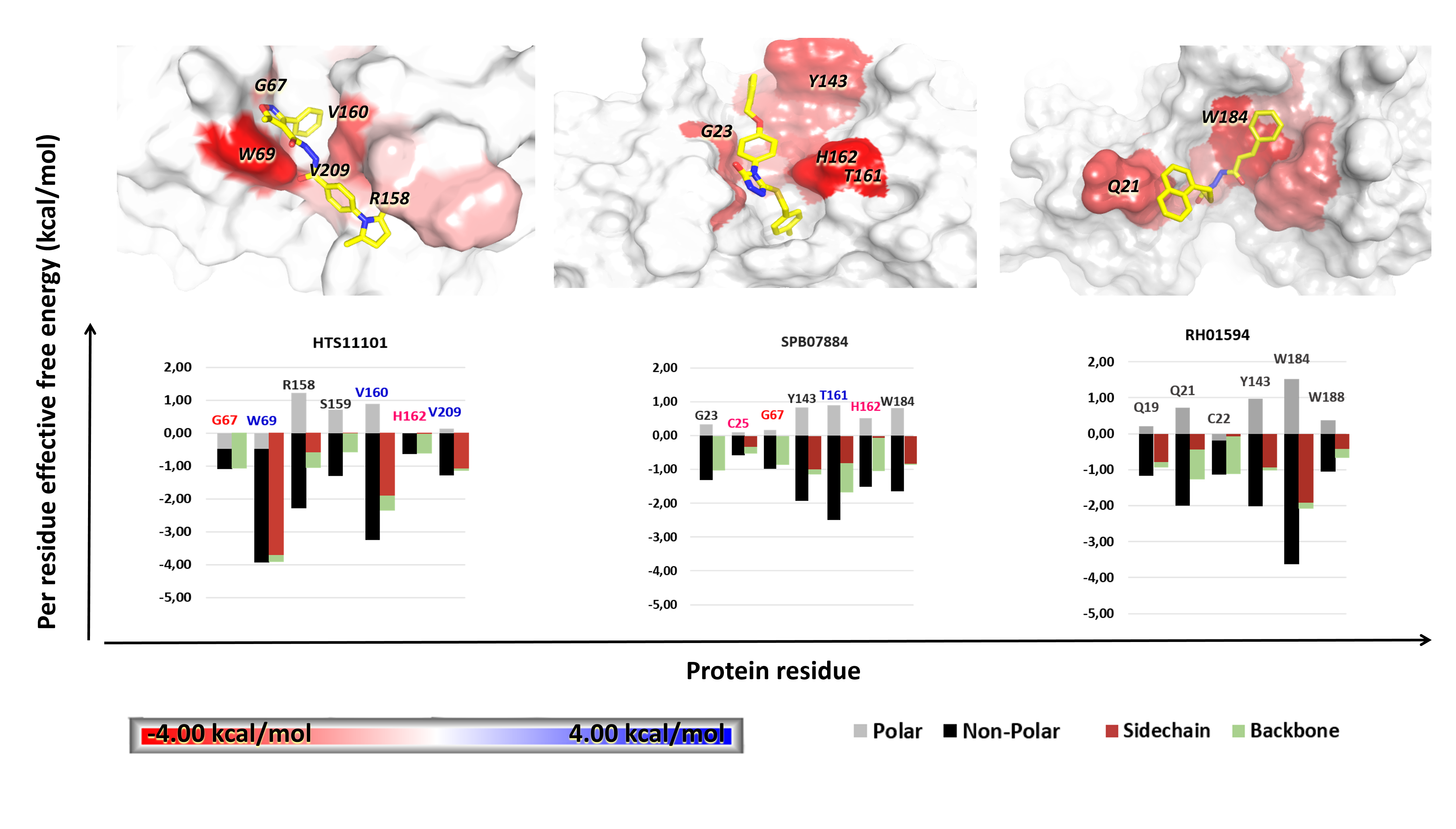

Supplement: S7 Fig — Bar graphs show the side chain, backbone, polar and non-polar contributions for each residue. Residue names are colored according to their location within S1 (pink), S2 (blue) and S3 (red) subsites. A structural representation of each complex interface is depicted as well. Interacting residues are colored according to energy value as shown in color scale. Hot/warm-spots are labeled in each case. (TIF) [file pntd.0003759.s007.tif]

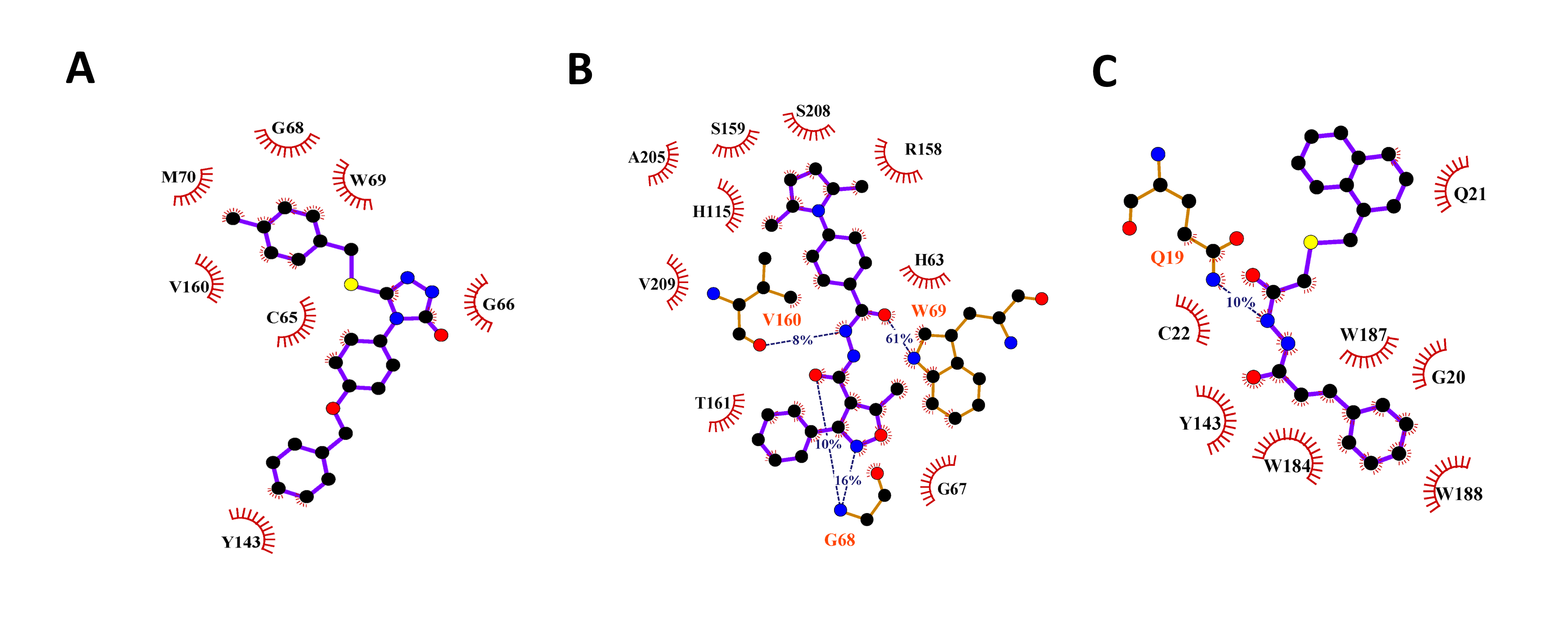

Supplement: S8 Fig — FhCL3 residues interacting with SPB07884 (A), HTS11101 (B), RH01594 (C). Ligands (violet) and protein residues involved in polar interactions (brown) are depicted in ball and stick representation. Hydrogen bonds (blue dashed lines) are shown together with their occupancy percent. Hydrogen donor and acceptor labels are shown in italic and bold styles, respectively. Residues establishing non-polar contacts are depicted as red semicircles. (TIF) [file pntd.0003759.s008.tif]
