# Supplementary material for: Insights into the Interactions of Fasciola hepatica Cathepsin L3 with a Substrate and Potential Novel Inhibitors through In Silico Approaches
Source: PLoS Negl Trop Dis. 2015 May 15;9(5):e0003759. doi: 10.1371/journal.pntd.0003759 (PMC4433193; doi:10.1371/journal.pntd.0003759)
Supplement: S1 Table — a Selected model. (DOCX) [file pntd.0003759.s009.docx]

| **Models** | **Procheck (%)** | **Verify_3D (%)** | **Errat** | **Qmean_Z-score** | **DOPE** |
| --- | --- | --- | --- | --- | --- |
| FHCL3_7 ^a^ | 100.00 | 91.82 | 93.10 | -0.07 | -24178.86 |
| FHCL3_6 | 99.50 | 90.45 | 87.00 | -0.48 | -24132.24 |
| FHCL3_15 | 100.00 | 90.45 | 89.90 | -0.02 | -24173.75 |
| FHCL3_12 | 99.00 | 94.09 | 94.08 | 0.30 | -24172.85 |
| FHCL3_13 | 99.00 | 95.00 | 88.67 | -0.28 | -24159.20 |
| FHCL3_14 | 100.00 | 94.09 | 88.67 | -0.06 | -24153.70 |
| FHCL3_2 | 99.50 | 90.45 | 85.24 | -0.44 | -24140.18 |
| FHCL3_4 | 100.00 | 94.09 | 92.12 | -0.29 | -24178.16 |
| FHCL3_3 | 99.00 | 91.36 | 87.20 | -0.25 | -24129.55 |
| FHCL3_1 | 100.00 | 95.45 | 89.95 | -0.27 | -24123.38 |
| FHCL3_9 | 100.00 | 94.09 | 87.56 | -0.54 | -24120.45 |
| FHCL3_5 | 100.00 | 92.27 | 91.54 | -0.28 | -24106.33 |
| FHCL3_11 | 100.00 | 93.18 | 93.30 | -0.27 | -24050.51 |
| FHCL3_8 | 99.50 | 95.00 | 85.07 | -0.24 | -23997.96 |
| FHCL3_10 | 100.00 | 92.27 | 89.27 | -0.11 | -23992.32 |
