# Supplementary material for: Insights into the Interactions of Fasciola hepatica Cathepsin L3 with a Substrate and Potential Novel Inhibitors through In Silico Approaches
Source: PLoS Negl Trop Dis. 2015 May 15;9(5):e0003759. doi: 10.1371/journal.pntd.0003759 (PMC4433193; doi:10.1371/journal.pntd.0003759)
Supplement: S2 Table — (DOCX) [file pntd.0003759.s010.docx]

| **Molecule ID** | **ΔS_vina_** | **SVM Score ^b^** | **Prediction** | **Probability Score** |
| --- | --- | --- | --- | --- |
| RH01594 | -2.6 | 0.021568546 | **Drug-like** | Low |
| RJC00878 | -2.6 | -0.26112832 | Non Drug-like | Low |
| HTS11101 | -2.4 | 0.05661755 | **Drug-like** | Low |
| NRB05245 | -1.9 | -0.13741202 | Non Drug-like | Low |
| HTS12701 | -1.8 | 0.30041088 | **Drug-like** | Low |
| BTB02457 | -1.8 | -0.2257229 | Non Drug-like | Low |
| RDR02526 | -1.8 | -0.088834568 | Non Drug-like | Low |
| RH00035 | -1.6 | -0.23411794 | Non Drug-like | Low |
| BTB03219 | -1.6 | 0.32685065 | **Drug-like** | Low |
| SEW01466 | -1.6 | -0.039958413 | Non Drug-like | Low |
| SPB07884 | -1.6 | 0.27531977 | **Drug-like** | Low |
| HTS08818 | -1.6 | -0.002361287 | Non Drug-like | Low |
